# The effect of pituitrin on postoperative outcomes in patients with pulmonary hypertension undergoing cardiac surgery: a study protocol for a randomized controlled trial

**DOI:** 10.3389/fcvm.2023.1269624

**Published:** 2024-01-03

**Authors:** Lingchen Kong, Meng Lv, Chang-long Qiao, Xia-xuan Sun, Wen-ya Du, Quan Li

**Affiliations:** ^1^ Department of Cardiovascular Surgery, The First Affiliated Hospital of Shandong First Medical University & Shandong Provincial Qianfoshan Hospital, Shandong Engineering Research Center for Health Transplant and Material, Jinan, Shangdong Province, China; ^2^ Shandong First Medical University & Shandong Academy of Medical Sciences, Jinan, China; ^3^ Anesthesiology Department, Shandong Provincial Hospital Affiliated to Shandong First Medical University, Jinan, Shandong Province, China; ^4^ Department of Anesthesiology, The First Affiliated Hospital of Shandong First Medical University, Jinan, Shandong Province, China; ^5^ Shandong Provincial Third Hospital, Cheeloo College of Medicine, Shandong University, Jinan, Shandong Province, China

**Keywords:** pituitrin, pulmonary hypertension, cardiac surgical procedures, vasopressor, perioperative medication

## Abstract

**Background:**

The vasoplegic syndrome is one of the major consequences of cardiac surgery. If pulmonary hypertension is additionally involved with vasoplegic syndrome, circulation management becomes much more complicated. According to previous studies, pituitrin (a substitute for vasopressin, which contains vasopressin and oxytocin) not only constricts systemic circulation vessels and increases systemic circulation pressure but also likely decreases pulmonary artery pressure and pulmonary vascular resistance. The aim of this study is to investigate whether pituitrin is beneficial for the postoperative outcomes in patients with pulmonary hypertension undergoing cardiac surgery.

**Methods and analysis:**

The randomized controlled trial will include an intervention group continuously infused with 0.04 U/(kg h) of pituitrin and a control group. Adult patients with pulmonary hypertension undergoing elective cardiac surgery will be included in this study. Patients who meet the conditions and give their consent will be randomly assigned to the intervention group or the control group. The primary outcome is the composite endpoint of all-cause mortality within 30 days after surgery or common complications after cardiac surgery. Secondary outcomes include the incidence of other postoperative complications, length of hospital stay, and so on.

**Discussion:**

Pituitrin constricts systemic circulation vessels, increases systemic circulation pressure, and may reduce pulmonary artery pressure and pulmonary vascular resistance, which makes it a potentially promising vasopressor during the perioperative period in patients with pulmonary hypertension. Therefore, evidence from randomized controlled trials is necessary to elucidate whether pituitrin influences outcomes in patients with pulmonary hypertension following cardiac surgery.

## Background

The vasoplegic syndrome after cardiopulmonary bypass is a prominent reason for early postoperative death and serious complications (1–3). The incidence of vasoplegic syndrome in patients undergoing cardiac surgery varies from 5% to 25%. Norepinephrine is currently considered as the first-line vasopressor agent for vasoplegic shock. Catecholamines commonly used in cardiac surgery may aggravate the condition of pulmonary hypertension (PH) (4, 5), while the use of drugs that reduce pulmonary hypertension, such as nitric oxide (NO), prostaglandins, and phosphodiesterase inhibitors, may worsen the vasoplegia after cardiopulmonary bypass (6).

Pituitrin is one of the alternative drugs for vasopressin. Vasopressin and oxytocin are two effective components in pituitrin, of which vasopressin is the main component, having a strong vasoconstrictive effect (7). Vasopressin exerts a non-catecholamine-related vasoconstrictive effect through several pathways, including modulation of adenosine triphosphate-sensitive K^+^ channel function and nitric oxide production, and enhancement of the vascular response to catecholamines. It can interact with V_1_ receptors distributed in the vascular smooth muscles, the pituitary gland, and the kidneys to exert a vasoconstrictive effect (8, 9). In addition, oxytocin can also bind to receptors distributed in the heart and vascular endothelium, and play a role by releasing atrial natriuretic peptide and nitric oxide (10). Therefore, pituitrin not only constricts systemic circulation vessels and increases systemic circulation pressure, it also likely reduces pulmonary artery pressure, pulmonary vascular resistance (11–13), atrial fibrillation, and acute renal injury (14–16). When pituitrin accumulates to a certain extent in the body, there are many adverse reactions, such as neurological symptoms including headache, disturbance of consciousness, irritability, and gastrointestinal symptoms, including nausea and vomiting, loss of appetite, and abdominal pain. It can also lead to myocardial ischemia and arrhythmias, causing symptoms of chest tightness and chest pain (17). Furthermore, hyponatremia, electrolyte abnormalities such as blood potassium, blood chloride, blood calcium, and blood magnesium may also occur.

At present, many studies have evaluated the role of vasopressin in the treatment of vasodilative syndrome. Hajjar et al. (18), Russell (19) , and other three randomized controlled trials (RCTs) (20–22) showed that the perioperative use of antidiuretic hormones may improve some clinical outcomes. In an expert consensus (23) in 2020, vasopressin was also recommended as the first-line medication for cardiac surgery. However, to our knowledge, no studies have explored the effect of pituitrin on the postoperative outcomes in patients with pulmonary hypertension after cardiac surgery.

This study aims to investigate the effect of pituitrin on the postoperative outcomes in patients undergoing cardiac surgery with pulmonary hypertension. Our hypothesis is that the use of pituitrin in patients with pulmonary hypertension undergoing cardiac surgery can reduce the postoperative mortality and the incidence of common complications after cardiac surgery. In addition, our hypothesis is that the application of pituitrin during the perioperative period of cardiac surgery has a certain impact on the incidence of other postoperative complications (postoperative infection, septic shock, respiratory system complications, etc.).

## Method and design

### Study design

This trial is a randomized, single-blind, parallel group, controlled trial. This study is registered on www.ClinicalTrials.gov (NCT05727618). The time schedule of the study is provided in Table 1. The study protocol is prepared according to the Standard Protocol Items: Recommendations for Interventional Trials (SPIRIT) checklist.

**Table 1:** Time schedule of enrollment, interventions, assessments, and visits for participants.

	Enrolment	Allocation	Post-allocation		Follow-up			
Timepoint	1	2	T0	T1	4	5	6	7
Eligibility screen	X							
Informed consent	X							
Allocation		X						
Interventions								
Group A			X	X				
Group B			X	X				
Assessments								
1. The composite endpoint of all-cause mortality 30 days after surgery or common complications after cardiac surgery								X
2. Incidence of postoperative infection, septic shock								X
3. Mechanical ventilation time								X
4. Incidence of respiratory complications, acute respiratory distress syndrome								X
5. Time to hemodynamic stability, use of dobutamine or other vasoactive drugs								X
6. Incidence of digital ischemia, acute mesenteric ischemia								X
7. Incidence of gastrointestinal complications								X
8. Incidence of acute myocardial infarction, new onset tachyarrhythmia								X
9. Incidence of vasodilatory shock, pulmonary embolism, low cardiac output syndrome								X
10. Incidence of delirium								X
11. Need for RRT, water poisoning, hyponatremia								X
12. Incidence of reentry to ICU								X
13. ICU and hospital length of stay								X
14. Serum antidiuretic hormone and peptide levels			X		X	X	X	

Post-allocation, from admission to the operation room to the beginning of the surgery. T0, admission to the operation room; T1, removing the aortic blocking forceps; 4, 4 h after the surgery; 5, 12 h after the surgery; 6, 24 h after the surgery; 7, 30 days after surgery; Group A, receives continuous intravenous infusion of pituitrin [Pituitrin is diluted to 0.5 U/ml and pumped intravenously at 0.04 U/(kg h)]; Group B, receives normal saline pumped at the same speed.

### Setting and recruitment

**Figure 1:**
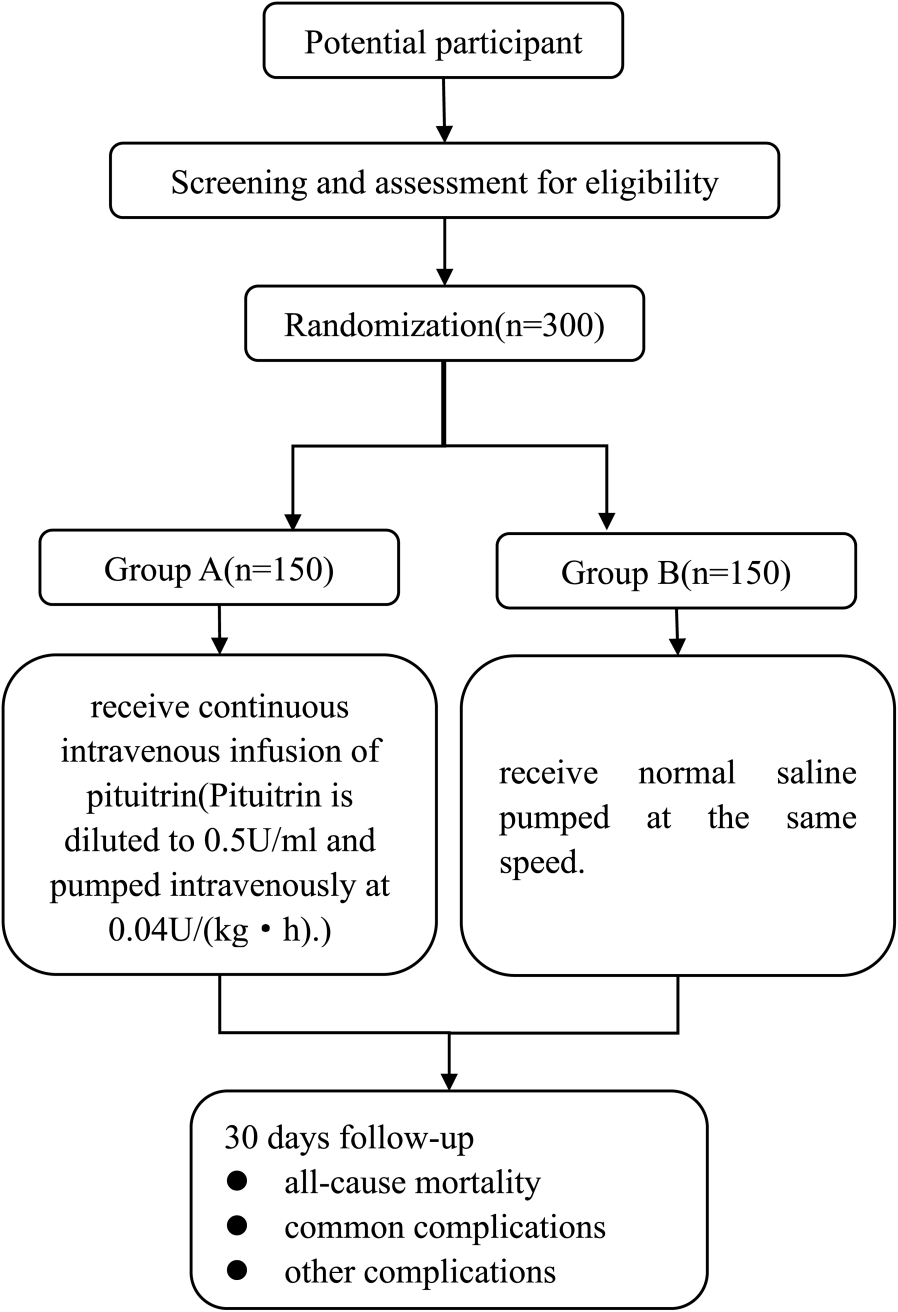
Flow diagram of enrollment, interventions, and assessment.

The screening and enrollment of the participants will be performed before the day of surgery. This study will be conducted in The First Affiliated Hospital of Shandong First Medical University & Shandong Provincial Qianfoshan Hospital, Jinan, China. It will include 300 adult patients with pulmonary hypertension who are scheduled for elective cardiac surgery under cardiopulmonary bypass. A total of 150 patients each will be recruited for the pituitrin group and the control group, respectively. A flow chart of the study is shown in Figure 1.

### Eligibility criteria

Patients, aged 18–80 years, with pulmonary hypertension [mean pulmonary artery pressure at rest ≥25 mmHg or pulmonary artery systolic pressure (PASP) ≥40 mmHg shown by right heart catheterization] who are scheduled to undergo elective heart surgery under cardiopulmonary bypass (congenital heart disease surgery, valve replacement, valvuloplasty, and heart transplantation) will be included.

The exclusion criteria include preoperative use of pituitrin or vasopressin; acute coronary syndrome; preoperative use of left ventricular assist devices; liver, thyroid, or adrenal diseases; severe lung diseases; diabetes mellitus; preoperative renal insufficiency [serum creatinine (SCR) increased ≥0.3 mg/dl (or ≥ 26.5 μmol/L); or if it is noted or speculated that the increase of SCR in the past 7 days exceeds 1.5 times the basic value; or 0.5 ml/kg urine volume per hour for 6 h]; severe carotid stenosis; preoperative stroke and mental disorder; difficulty in communicating and cooperating; suffering from peripheral vascular disease; hypersensitivity or allergy to vasopressin or pituitrin; severe hyponatremia (Na^+^ < 130 mmol/L); acute mesenteric ischemia; pregnancy; malignancy; emergency surgery or reoperation; and participation in other clinical trials during the past 3 months.

### Informed consent procedure

The investigators will provide the subjects or their legal representatives with an easily understood informed consent form approved by the ethics committee, and give the subjects or their legal representatives sufficient time to consider the study. Participants will be required to sign an informed consent form before participating in the study. They will be provided with a clear explanation of the purpose, process, risks, and benefits of this study, and all questions asked by the participants with the intention of confirming their understanding of the study, the potential risks, and benefits involved in the study, as well as their privileges as study subjects, will be answered before they sign the consent papers. Informed consent should be obtained prior to any research evaluation or procedures, and before any private information is recorded. During the study period, participants will have an opportunity to agree to continue using the study protocol. We will also request that subjects to invite their family and friends to participate in an initial discussion about the study so that all individuals involved in deciding whether to enroll in the study have adequate information. When the results of the study are published, no information about the subjects will be disclosed.

### Randomization and allocation concealment

After the participants sign the informed consent, they will be randomly assigned into the two groups according to a computer-generated random number table. The study nurse who is responsible for random number generation will put the allocation information in an opaque envelope and assign it to the anesthesiologist responsible for the intraoperative management of the participants after the participants enter the operation room. The study drug will also be prepared by the study nurse. To ensure the safety of patients, the anesthesiologist responsible for the intraoperative management will be informed of the treatment allocation. Participants and outcome assessors will be blinded to treatment allocation.

### Procedure for unblinding

To ensure the safety of the subjects, in cases of emergency, if serious adverse events (such as severe hyponatremia) occur and it is impossible to judge whether they are related to the test drug, excessive drug use, or serious drug interaction with concomitant drug use, the blindness can be uncovered in advance when it is urgent to decide the rescue plan according to the procedures specified in the test plan. Once the case number is opened, the trial will be suspended, and the researcher shall record the reason for suspension in the case report form.

### Intervention

After removing the aortic cross clamp, the study drug (pituitrin diluted to 0.5 U/ml or equal volume of normal saline) will be continuously administered. Group A will receive continuous intravenous infusion of pituitrin [pituitrin is diluted to 0.5 U/ml with an initial infusion rate at 0.04 U/(kg h)], and Group B will receive intravenous infusion of normal saline at the same speed. The anesthesiologist will decide whether to use other vasoactive drugs and will adjust the dosage according to the hemodynamic status of the patient. When mean arterial pressure (MAP) is >70 mmHg, the pump speed of the norepinephrine will be first reduced. Only if the dosage of norepinephrine is reduced to 0.05 μg/(kg min), the study drug will be decreased gradually until it is completely terminated.

### Assessments

The age, gender, and medical history of the participants will be recorded at baseline. Investigators will screen the subjects according to the inclusion and exclusion criteria one day before the surgery. The subjects will be followed up within 30 days after operation. From the first day to the third day after surgery, the participants will be followed up by a researcher at 8 am and 8 pm each day, asking about the recovery of the participants after surgery, and reviewing the relevant laboratory tests and auxiliary tests of the participants. Our researchers will conduct a telephone follow-up of the subjects 30 days after surgery to inquire about their recovery and the occurrence of postoperative complications. The follow-up will last until 30 days after surgery. If the patient is hospitalized for more than 30 days after surgery, follow-up will be performed until the day of discharge.

### Outcomes

The primary outcome is the composite endpoint of all-cause mortality 30 days after surgery or common complications after cardiac surgery (stroke, requiring mechanical ventilation for more than 48 h, deep sternal wound infection, reoperation, extracorporeal membrane oxygenation, atrial fibrillation, or acute renal injury) (12).

Stroke is an acute episode of focal dysfunction of the brain, retina, or spinal cord lasting longer than 24 h, or of any duration if imaging [computed tomography (CT) or magnetic resonance imaging (MRI)] or autopsy show focal infarction or hemorrhage relevant to the symptoms. Deep sternal wound infection is defined by infection of the sternal wound with positive findings on cultures or suggestive findings on thorax computed tomographic scan. Acute renal failure is defined as a new requirement for hemodialysis, an increase in serum creatinine to more than 2.0 mg/dl, or double the most recent preoperative creatinine level (24, 25).

Secondary outcomes include the incidence of postoperative infection, septic shock, mechanical ventilation time, respiratory complications (including atelectasis, pleural effusion, diaphragmatic dysfunction, prolonged mechanical ventilation, pneumonia, pneumothorax, acute respiratory distress syndrome, etc.); hemodynamic effects (time to hemodynamic stability, use of dobutamine or other vasoactive drugs); incidence of digital ischemia, acute mesenteric ischemia, gastrointestinal complications; incidence of acute myocardial infarction, new onset tachyarrhythmia, water intoxication, vasodilatory shock; incidence of pulmonary embolism, low cardiac output syndrome, acute respiratory distress syndrome, delirium; need for renal replacement therapy (RRT), incidence of hyponatremia; incidence of readmission to ICU, ICU and hospital length of stay, serum antidiuretic hormone, and peptide levels immediately after entering the operating room and 4, 12, and 24 h after surgery.

### Adverse events

Clinical adverse events may occur during the treatment of subjects. Once adverse events (including important adverse events) occur, the occurrence time, clinical manifestation, treatment process and duration, outcome, and relationship with drugs should be recorded in detail on the case report form. In case of abnormal laboratory test, the patient should be followed up until the test results return to normal, or to the level before medication, or it is determined that it is not related to the test drug. In case of serious adverse events, the serious adverse event form should be filled out and reported to the ethics committee within 24 h. All adverse events should be followed up until they are properly resolved or the condition is stable.

## Statistical analysis

### Sample size

The study is intended to be a superiority study, and the ratio between the two groups will be 1:1; single side *α *=* *0.025; statistical power = 0.80, optimality limit will be 5% (18). Based on the reference data, the event rate of the control group is set to be 0.5, while the event rate of the study drug group is set at 0.35. It is estimated that the drop-out rate will be 10%, and 150 cases in each group can be considered.

### Plan of data analysis

For normally distributed data, the mean and standard deviation will be used, and alternatively, for non-normally distributed data, the median and interquartile range (IQR) will be implemented. The normality of continuous variables will be assessed using the Kolmogorov–Smirnov test. Categorical variables will be presented in terms of frequency and percentage. Independent sample *t*-test will be used for the data conforming to normal distribution, otherwise non-parametric test (Mann–Whitney *U*) will be used. In addition, time-related variables will be analyzed with the log-rank and Kaplan–Meier methods. Each individual outcome of a composite outcome will be analyzed separately, and subgroup analysis will be performed according to different surgical types. The mortality of different time points will be analyzed using Cox analysis. Logistic regression will be utilized to consider potential confounding factors. *p* < 0.025 is considered statistically significant. All data will be analyzed by using SPSS (25.0).

### Ethics and dissemination

The scientific research branch of The First Hospital affiliated of Shandong First Medical University has meticulously reviewed the proposed study, providing feedback to the design and methodology of the study. Furthermore, the big data center also scrutinized the proposal.

To safeguard the rights of the patients, the research team will provide them and their families with information sheets detailing the study's objectives and procedures. Eligible participants who express interest in participation must sign a consent form that outlines the steps involved in the pre- and post-intervention process, as well as the potential benefits and risks of intervention studies. Participants will receive confirmation of confidentiality and protection of collected information. The results of the study will not be disseminated to the study participants unless one of the participants wants to know his own results.

### Publication and dissemination plan

The results of this study will be disseminated in peer-reviewed international medical journals. Further exploration employing biological specimens for supplementary research has yet to be determined.

### Protocol amendments

If there is a protocol revision that requires ethical approval, the protocol will not be modified until the revised and revised informed consent form and revised case report form are reviewed and approved/favorable comments are obtained from the ethics committee. Any significant modification will be communicated to the principal investigators involved.

### Confidentiality and access to data

The staff and investigators will take all necessary measures to safeguard the privacy and informed consent of study participants, adhering to the Data Protection Act of 2018 (26). The case report forms will solely collect the minimum required information for the purposes of the trial, which will be securely stored in locked rooms or cabinets. Access to the data will be limited only to authorized trial staff, investigators, and pertinent regulatory authorities. Computer-based data, including the trial database, will be encrypted and password-protected, with secure storage on a dedicated web server. Access will be restricted by user identifiers and passwords. All details concerning the trial within the medical records or hospital notes of the participants will be treated with the same level of confidentiality as other sensitive medical information. Electronic data will be backed up every 24 h to both local and remote media in encrypted format, thus ensuring their safety and security. The minimum retention time for clinical trial records is 5 years.

## Discussion

Inhalation of NO can selectively decrease pulmonary artery pressure, and it is the preferred treatment method for severe postoperative PH in many centers. However, there is no inhaled NO system approved for medical use in China. Aerosolized vasodilators are commonly used in treatment for perioperative PH (27, 28), they can significantly reduce pulmonary vascular resistance and have minimal impact on systemic hemodynamics. But studies have shown that inhaling nebulized vasodilators for pulmonary hypertension does not have a significant benefit in reducing mortality (29).

Pituitrin can not only constrict systemic circulation vessels and increase systemic circulation pressure (8, 9, 30), it can also have small impact on pulmonary artery pressure and pulmonary vascular resistance (11–13). Therefore, pituitrin may play an important role in cardiac surgery for patients with pulmonary hypertension. Despite this, the impact of pituitrin on patient prognosis after perioperative application has not been reported in relevant studies, underscoring the need for randomized controlled trials to provide essential evidence. The results of this RCT will fill the gap of the effect of pituitrin on the prognosis in patients undergoing cardiac surgery, and provide new ideas for optimizing the management of the patients with pulmonary hypertension and improving the prognosis of the vasoplegic syndrome, which has significant clinical value.

Our study has several strengths. First, to our knowledge, this is the first, large sample size, randomized controlled trial to investigate the effect of pituitrin on the postoperative outcomes of the patients undergoing cardiac surgery. Second, the study is well designed, and the methodology of this study is of high quality. Third, study procedure and rescue treatment are thoughtful to ensure the safety of the participants.

However, there are also several limitations in this study. First, this is a single-center study, and the external applicability of our findings may be limited. Second, this is a single-blind study to ensure the safety of the participants; therefore, it might raise the performance bias.

## Conclusion

Given that pituitrin is widely available and cost-effective, it may represent a viable means of reducing the risks of postoperative complications in patients undergoing cardiac surgery with pulmonary hypertension.
